# Silibinin protects against osteoarthritis through inhibiting the inflammatory response and cartilage matrix degradation *in vitro* and *in vivo*

**DOI:** 10.18632/oncotarget.20587

**Published:** 2017-08-24

**Authors:** Wenhao Zheng, Zhenhua Feng, Yiting Lou, Chunhui Chen, Chuanxu Zhang, Zhenyu Tao, Hang Li, Liang Cheng, Xiaozhou Ying

**Affiliations:** ^1^ Department of Orthopaedic Surgery, The Second Affiliated Hospital and Yuying Children’s Hospital of Wenzhou Medical University, Wenzhou 325000, China

**Keywords:** osteoarthritis, chondrocyte, silibinin, inflammation, NF-κB

## Abstract

Osteoarthritis (OA) is a degenerative joint disease characterized by cartilage degradation and inflammation. Silibinin, a polyphenolic flavonoid derived from fruits and seeds of *Silybum marianum*, has been reported to possess various potent beneficial biological effects, such as antioxidant, anti-cancer, hepatoprotective and anti-inflammatory activities. However, the anti-inflammatory effects of silibinin on OA have not been reported. This study aimed to assess the effects of silibinin on OA both *in vitro* and *in vivo*. In this study, we found that silibinin significantly inhibited the nterleukin-1β (IL-1β)-induced production of nitric oxide (NO), prostaglandin E2 (PGE2), tumor necrosis factor-α (TNF-α) and IL-6, expression of cyclooxygenase2 (COX-2), inducible nitric oxide synthase (iNOS), matrix metalloproteinase-1 (MMP-1), MMP-3, MMP-13, a disintegrin and metalloproteinase with thrombospondin motifs-4 (ADAMTS-4) and ADAMTS-5, degradation of aggrecan and collagen-II in human OA chondrocytes. Furthermore, silibinin dramatically suppressed IL-1β-stimulated phosphatidylinositol 3 kinase/ protein kinase B (PI3K/Akt) phosphorylation and nuclear factor-kappa B (NF-kB) activation in human OA chondrocytes. In addition, treatment of silibinin not only prevented the destruction of cartilage and the thickening of subchondral bone but also relieved synovitis in mice OA models. Also, the immunohistochemistry results showed that silibinin significantly decreased the expression of MMP-13 and ADAMTS-5 and increased the expression of collagen-II and aggrecan in mice OA. Taken together, these results suggest that silibinin may be a potential agent in the treatment of OA.

## INTRODUCTION

Osteoarthritis (OA) is a multifactorial degenerative joint disorder associated with structural changes of joint tissues including cartilage destruction, subchondral bone remodeling as well as synovial inflammation in the elderly [1]. The prevalence of OA increases with age [2]. Previous studies showed that about 12% of western aging population are suffering from OA and 25% people aged over 55 have an episode of persistent knee pain [3]. However, the aetiology of OA remains unknown, inflammation and inflammatory cytokines have been reported to exert critical roles in the initiation and progression of OA [4]. It has been known that excess production of the inflammatory cytokines play vital roles in the development of OA [5]. Among these cytokines, interleukin-1β (IL-1β) has been shown to play a pivotal role in OA as it contributes to cartilage matrix degradation through inducing the expression of matrix metalloproteinases (MMPs) and a disintegrin and metalloproteinase with thrombospondin motifs (ADAMTS), which results in decreased synthesis of collagen and proteoglycan during the pathogenesis of OA [6]. Additionally, stimulating of chondrocytes with IL-1β could induce the release of inflammatory mediators nitric oxide (NO) and prostaglandin E2 (PGE2) [7, 8]. Overproduction of NO and PGE2 has been considered to be closely related with the clinical manifestations of OA [9]. Therefore, the inhibition of IL-1β and IL-1β-induced inflammatory mediators are suggested as a potential strategy to treat OA.

Silibinin is the main active component of silymarin, a polyphenolic flavonoid derived from fruits and seeds of *Silybum marianum*, has been reported to possess several potent beneficial biological effects such as antioxidant [10], anti-cancer [11], hepatoprotective [12] and anti-inflammatory activities [13]. Previous studies showed that silibinin inhibited the production of pro-inflammatory cytokines through inhibition of nuclear factor-kappa B (NF-κB) signaling pathway in HMC-1 human mast cells [14]. In addition, silibinin significantly inhibited IL-1β-induced production of pro-inflammatory mediators and attenuated NF-κB nuclear translocation in canine hepatocytes [15]. Silibinin was also found to suppress the lipopolysaccharides (LPS)-induced production of IL-1β and PGE2 in isolated mouse peritoneal macrophages and RAW 264.7 cells [16]. Furthermore, *in vivo* study, silibinin had the ability to inhibit airway inflammatory cell recruitment and peribronchiolar inflammation and reduce the production of various cytokines in bronchoalveolar fluid via down-regulation of NF-κB activity in a mouse ovalbumin (OVA)-induced asthma model [17]. However, the anti-inflammatory effect of silibinin on OA is still unclear. Consequently, in the current study, we investigated the anti-inflammatory effect and the potential mechanism of silibinin on IL-1β-stimulated human OA chondrocytes *in vitro* as well as the protective role of silibinin in mice OA models *in vivo*.

## RESULTS

### Effect of silibinin on human OA chondrocyte viability

**Figure 1:**
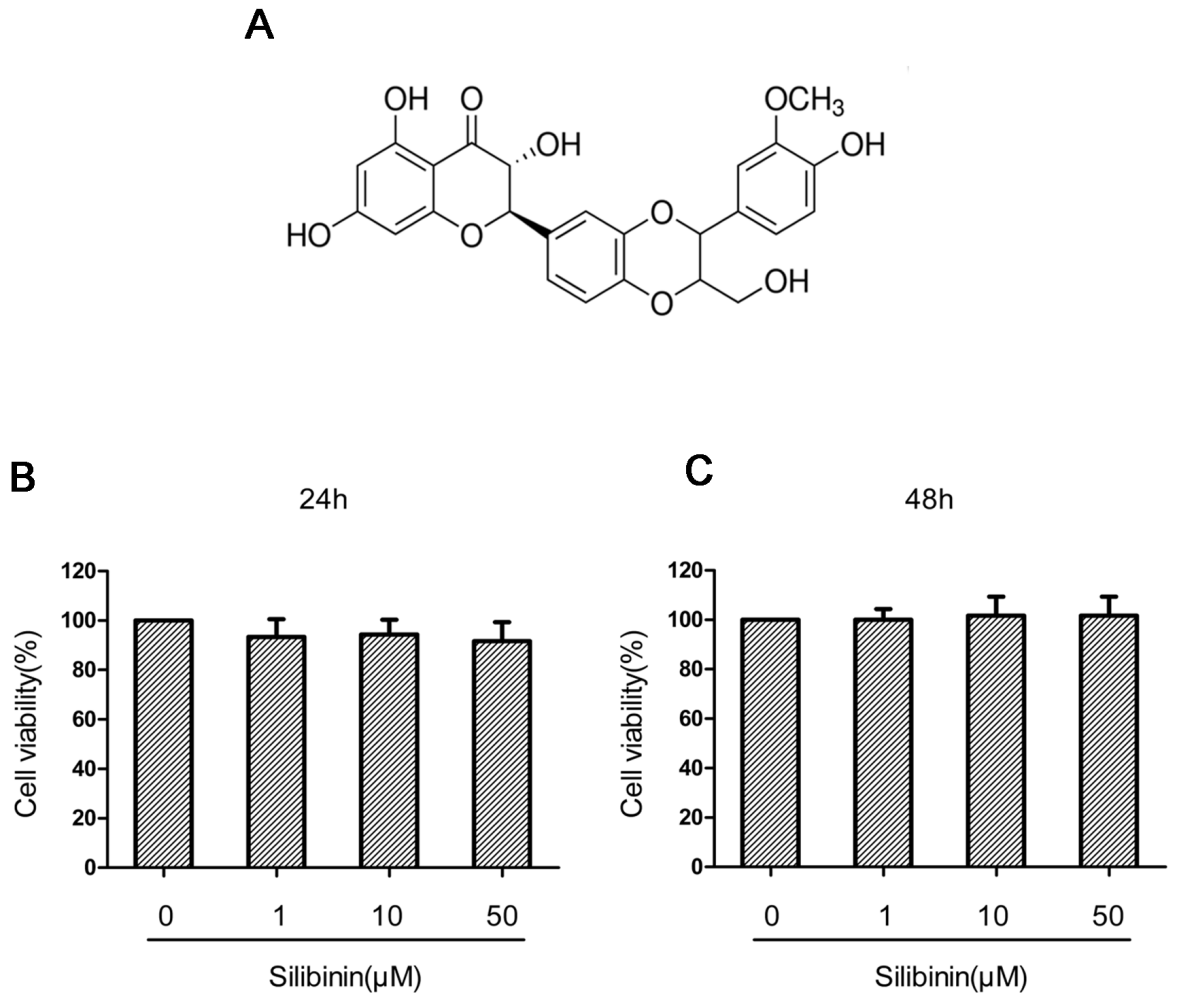
The chemical structure of silibinin and effect of silinibin on human OA chondrocyte viability The chemical structure of silibinin was shown in **(A).** The cells were cultured with increasing concentrations of silibinin (0, 1, 10 or 50μM) for 24 h and 48h. The cell viability was determined by CCK-8 assay **(B, C)**. The values are mean ± SD. ^*^ p < 0.05, ^**^ p < 0.01 compared with control group, n=6.

The effect of silibinin on the viability of chondrocytes was determined by CCK-8 assay. The chondrocytes were cultured with increasing concentrations of silibinin (0, 1, 10, 50μM) for 24h and 48h, followed by the CCK-8 analysis. The results showed that silibinin at the concentration range of 1–50 μM did not have cytotoxic effects on human OA chondrocytes (Figure 1B-1C). Consequently, silibinin (1, 10, 50μM) were used in the subsequent experiments.

### Effect of silibinin on IL-1β-induced NO, PGE2, TNF-α and IL-6 production in human OA chondrocytes

**Figure 2:**
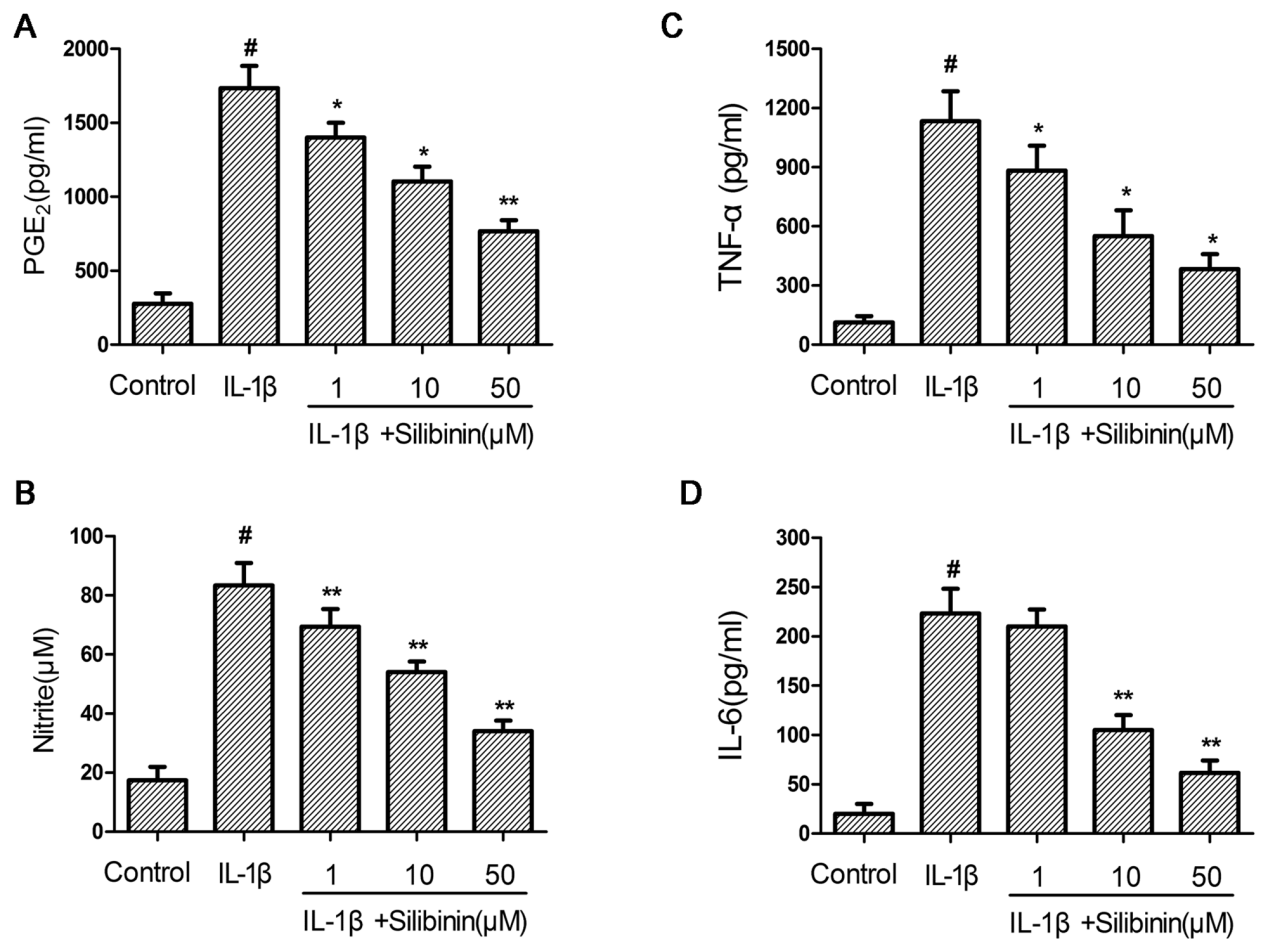
Effect of silibinin on IL-1β-induced NO, PGE2, TNF-α and IL-6 production in human OA chondrocytes Human OA chondrocytes were pretreated for 2 h with various concentrations of silibinin (1, 10, 50 μM) and then stimulated or not stimulated with IL-1β (10 ng/ml) for 24 h. The nitrite levels in the culture medium were assessed by Griess reaction **(B)**. The levels of PGE2, TNF-α and IL-6 were determined using ELISA **(A,C,D)**. The values are mean ± SD. ^#^p < 0.05 compared with control group, ^*^p < 0.05, ^**^p< 0.01 compared with IL-1β group. n=6.

First, the effect of silibinin on IL-1β-induced NO, PGE2, TNF-α and IL-6 production was detected. As shown in Figure 2A-2D, the production of NO, PGE2, TNF-α and IL-6 increased significantly in chondrocytes compared with the control group after IL-1β treatment. However, silibinin inhibited IL-1β-induced NO, PGE2, TNF-α and IL-6 production in a dose-dependent manner.

### Effect of silibinin on IL-1β-induced iNOs and COX-2 expression in human OA chondrocytes

**Figure 3:**
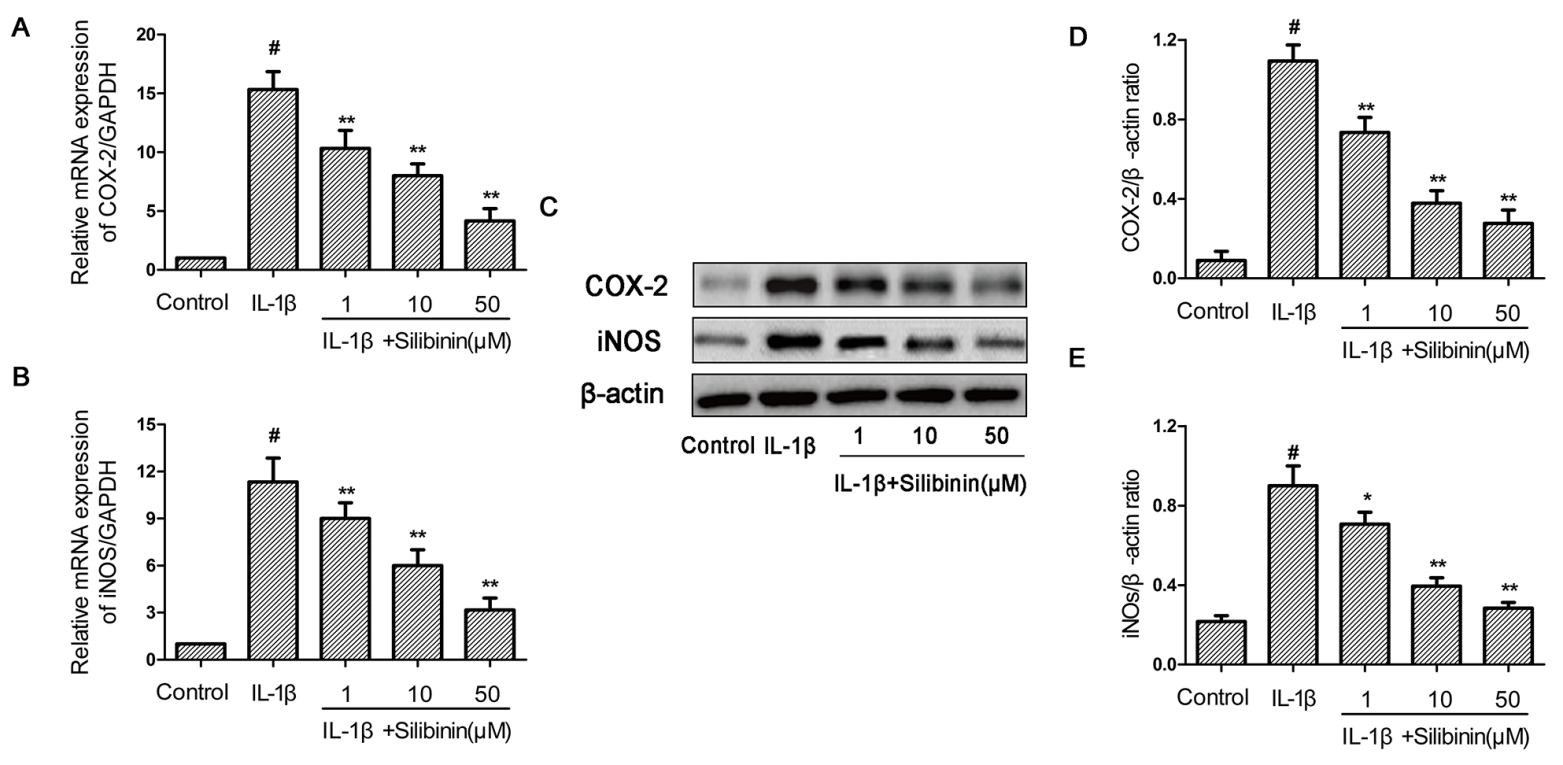
Effect of silibinin on IL-1β-induced iNOs and COX-2 expression in human OA chondrocytes Human OA chondrocytes were pretreated for 2 h with various concentrations of silibinin (1, 10, 50 μM) and then stimulated or not stimulated with IL-1β (10 ng/ml) for 24 h. The mRNA expression levels of iNOs and COX-2 **(A,B)** were assayed by qRT-PCR. The protein expression levels of iNOs and COX-2 were determined by Western blot and quantification analysis **(C-E)**. The values are mean ± SD. ^#^p < 0.05 compared with control group, ^*^p < 0.05, ^**^p< 0.01 compared with IL-1β group. n=6

Subsequently, we investigated the effect of silibinin on the expression of iNOS and COX-2 in human chondrocytes. As shown in Figure 3A-3B, IL-1β-stimulation of human OA chondrocytes resulted in significant up-regulation of iNOs and COX-2 mRNA expression compared to the control group. Whereas, silibinin dose-dependently inhibited the mRNA expression of iNOs and COX-2 induced by IL-1β. Consistent with the results of mRNA, the protein expression of iNOs and COX-2 induced by IL-1β were also remarkably suppressed by treatment of silibinin. (Figure 3C-3E)

### Effect of silibinin on IL-1β-induced MMP-1, MMP-3, MMP-13, ADAMTS-4 and ADAMTS-5 expression in human OA chondrocytes

**Figure 4:**
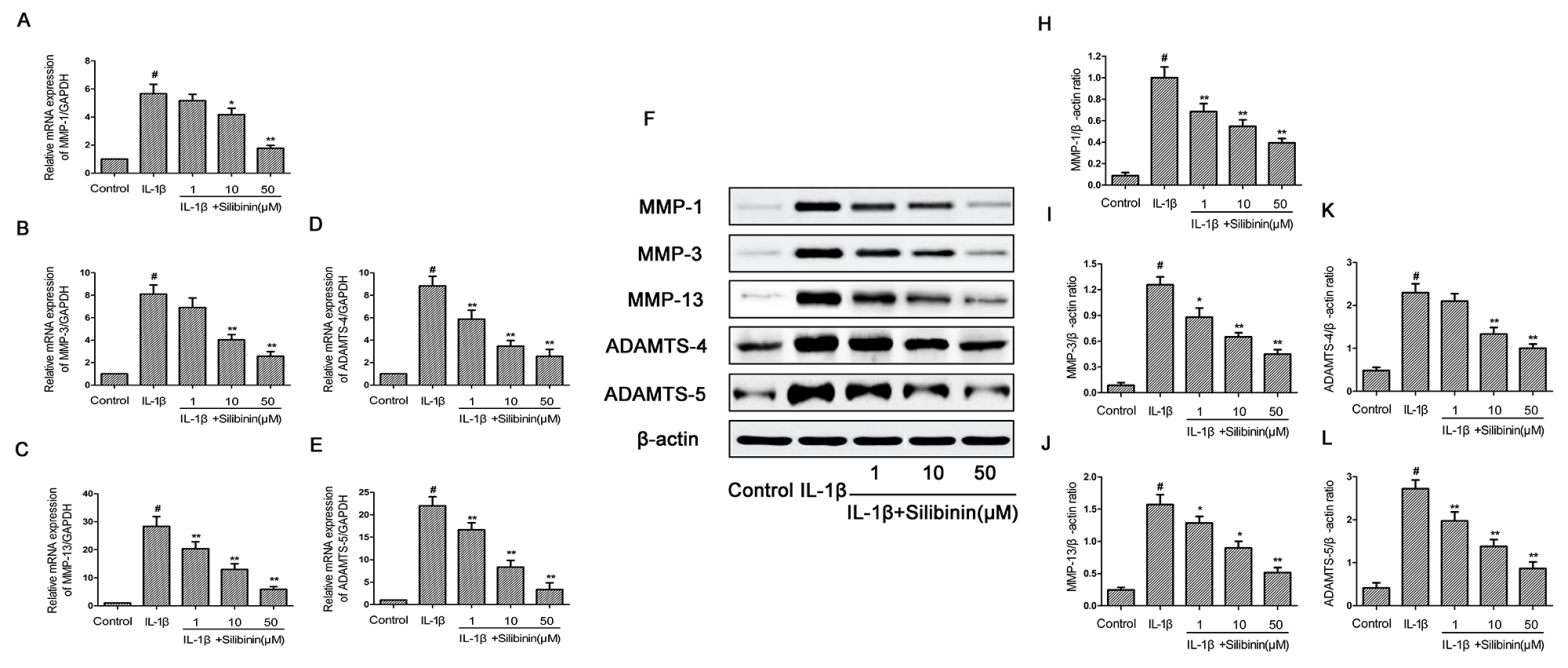
Effect of silibinin on IL-1β-induced MMP-1, MMP-3, MMP-13, ADAMTS-4 and ADAMTS-5 expression in human OA chondrocytes Human OA chondrocytes were pretreated for 2 h with various concentrations of silibinin (1, 10, 50 μM) and then stimulated or not stimulated with IL-1β (10 ng/ml) for 24 h. The mRNA expression levels of MMP-1 **(A)**, MMP-3 **(B)**, MMP-13 **(C)**, ADAMTS-4 **(D)** and ADAMTS-5 **(E)** were assayed by qRT-PCR. The protein expression levels of MMP-1, MMP-3, MMP-13, ADAMTS-4 and ADAMTS-5were determined by Western blot and quantification analysis **(F-L)**. The values are mean ± SD. ^#^p < 0.05 compared with control group, ^*^p < 0.05, ^**^p< 0.01 compared with IL-1β group. n=6

Next, we analyzed the effect of silibinin on MMP-1, MMP-3, MMP-13, ADAMTS-4 and ADAMTS-5 expression in human OA chondrocytes. As shown in Figure 4A-4E, chondrocytes showed obvious up-regulation of the mRNA expression of MMP-1, MMP-3, MMP-13, ADAMTS-4 and ADAMTS-5 after IL-1β stimulation in contrast with the control group. However, treatment of silibinin markedly reduced the mRNA up-regulation of MMP-1, MMP-3, MMP-13, ADAMTS-4 and ADAMTS-5. In line with the results of mRNA, treatment with silibinin apparently blocked the protein expression of MMP-1, MMP-3, MMP-13, ADAMTS-4 and ADAMTS-5.(Figure 4F-4L)

### Effect of silibinin on IL-1β-induced aggrecan and collagen-II degradation in human OA chondrocytes

**Figure 5:**
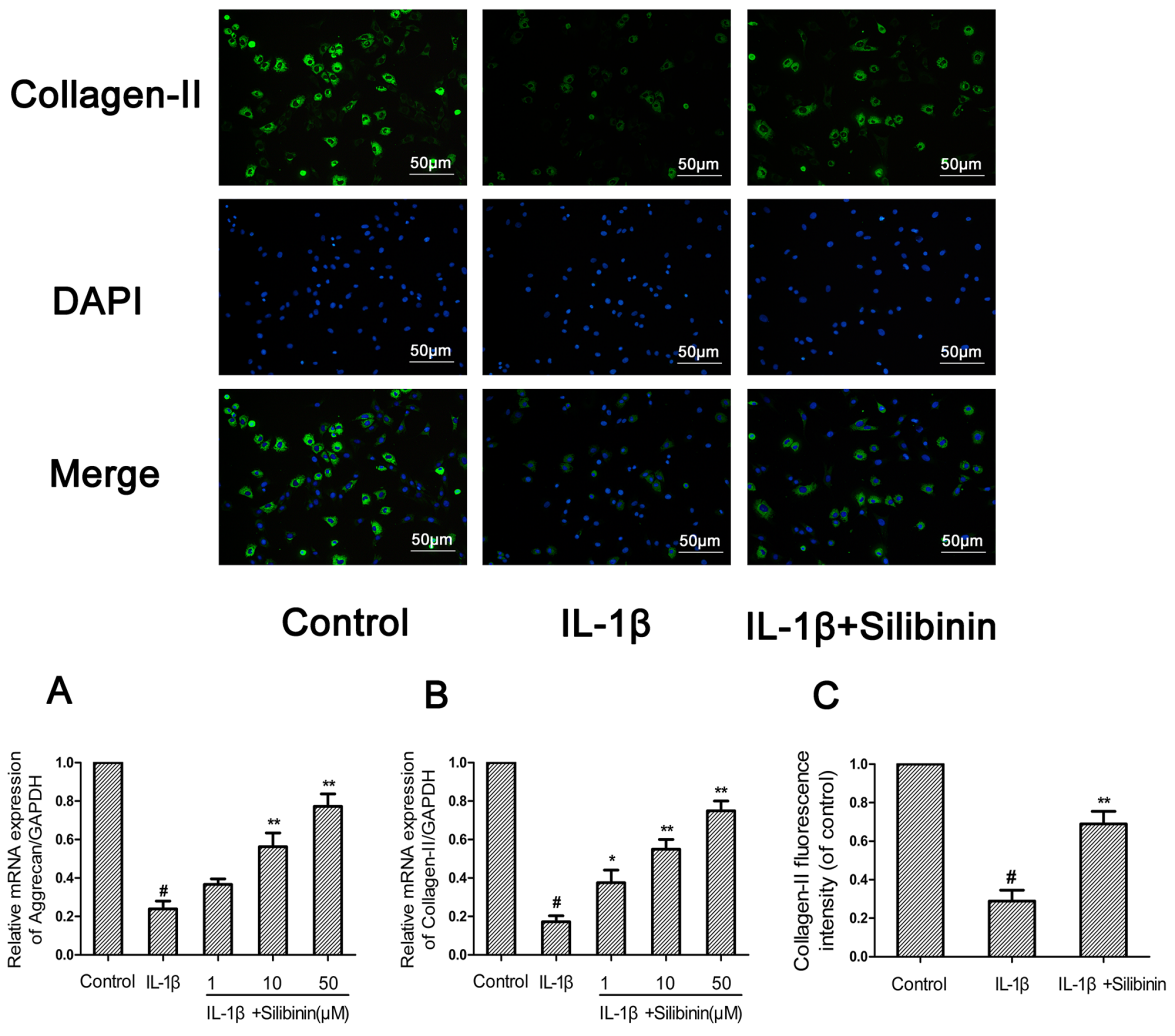
Effect of silibinin on IL-1β-induced aggrecan and collagen-II degradation in human OA chondrocytes Human OA chondrocytes were pretreated for 2 h with silibinin (1, 10, 50 μM) and then stimulated or not stimulated with IL-1β (10 ng/ml) for 24 h. The mRNA expression levels of aggrecan **(A)** and collagen-II **(B)** were assayed by qRT-PCR. The protein expression levels of collagen-II were determined by immunofluorescence and quantification analysis **(C)**. The values are mean ± SD. ^#^p < 0.05 compared with control group, ^*^p < 0.05, ^**^p < 0.01 compared with IL-1β group. n=6

Then, we investigated the effect of silibinin on IL-1β-induced aggrecan and collagen-II degradation in human OA chondrocytes. As shown in Figure 5A-5B, chondrocytes revealed an obvious down-regulation of the mRNA expression of aggrecan and collagen-II after IL-1β stimulation. On the contrary, silibinin clearly inhibited the mRNA down-regulation of aggrecan and collagen-II. Moreover, the immunofluorescence results suggest silibinin signally suppressed the protein degradation of collagen-II, which keeps in accord with the results of qRT-PCR.(Figure 5C)

### Effect of silibinin on IL-1β-induced NF-kB activation in human OA chondrocytes

**Figure 6:**
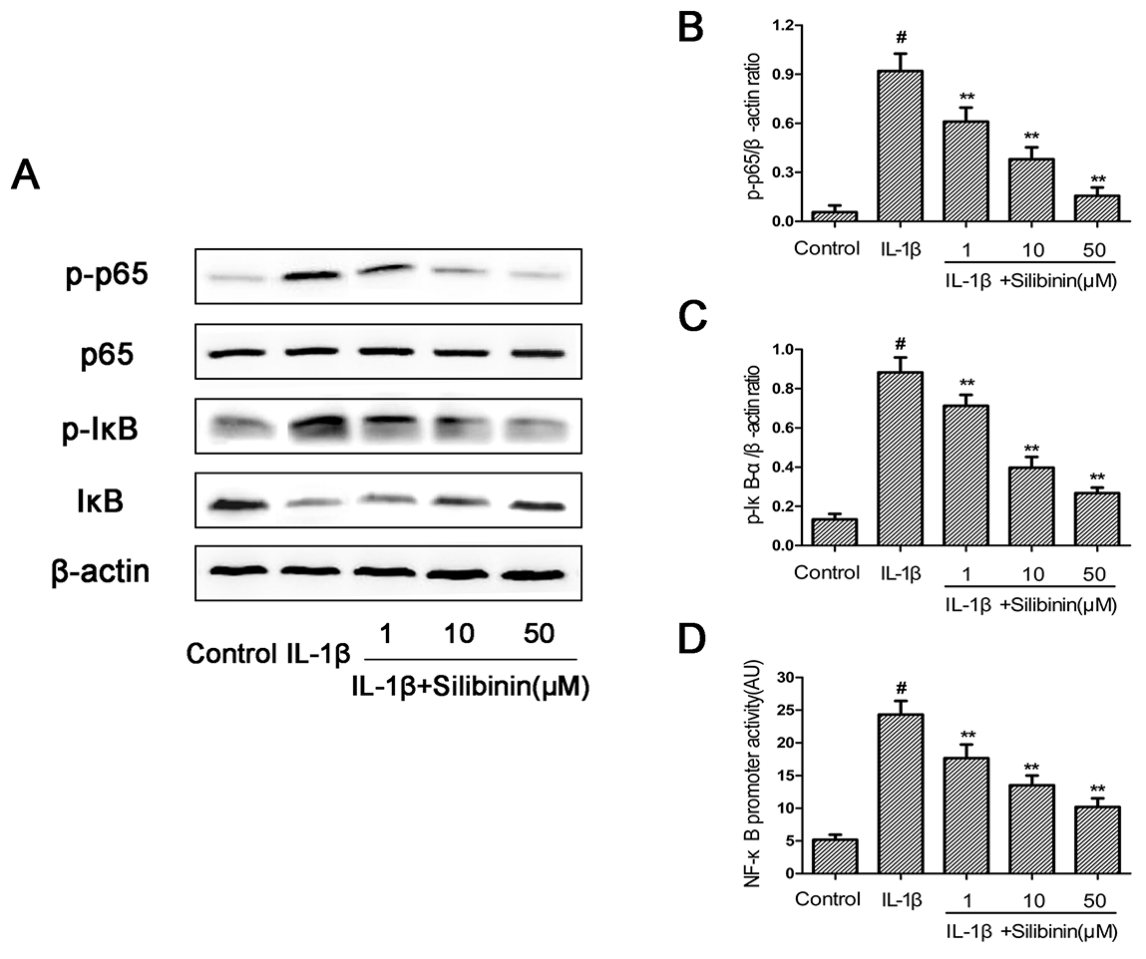
Effect of silibinin on IL-1β-induced NF-kB activation in human OA chondrocytes Chondrocytes were pretreated with silibinin (1, 10, 50 μM) for 2h, followed by stimulation with or without IL-1β (10 ng/ml) for 1 h. The protein expression levels of p65,p-p65,IκB and p-IκB were determined by Western blot and quantification analysis **(A-C)**. Chondrocytes were transfected with NF-kB luciferase lipofectamine and the NF-kB promoter activity was determined by luciferase assay kit **(D)**. The values are mean ± SD. ^#^p < 0.05 compared with control group, ^*^p < 0.05, ^**^p< 0.01 compared with IL-1β group, n=6

To further elucidate the mechanism underlying the anti-inflammatory effect of silibinin, Western blot and NF-kB-dependent gene reporter assay were performed to study changes in NF-κB signaling pathway. As shown in Figure 6, IL-1β stimulation significantly induced the phosphorylation of NF-κB p65 and IκBα in chondrocytes. Moreover, stimulation of chondrocytes with IL-1β resulted in conspicuous degradation of IκBα. In contrast, silibinin exhibited a concentration-dependent inhibitory effect on IL-1β-induced NF-kB activation and IkBα degradation in human OA chondrocytes (Figure 6A-6C). On the other side, the results of luciferase assays showed that silibinin significantly reduced NF-kB promoter luciferase activity induced by IL-1β in a dose dependent manner (Figure 6D).

### Silibinin inhibited IL-1β-induced nuclear-translocation of NF-κB p65

**Figure 7:**
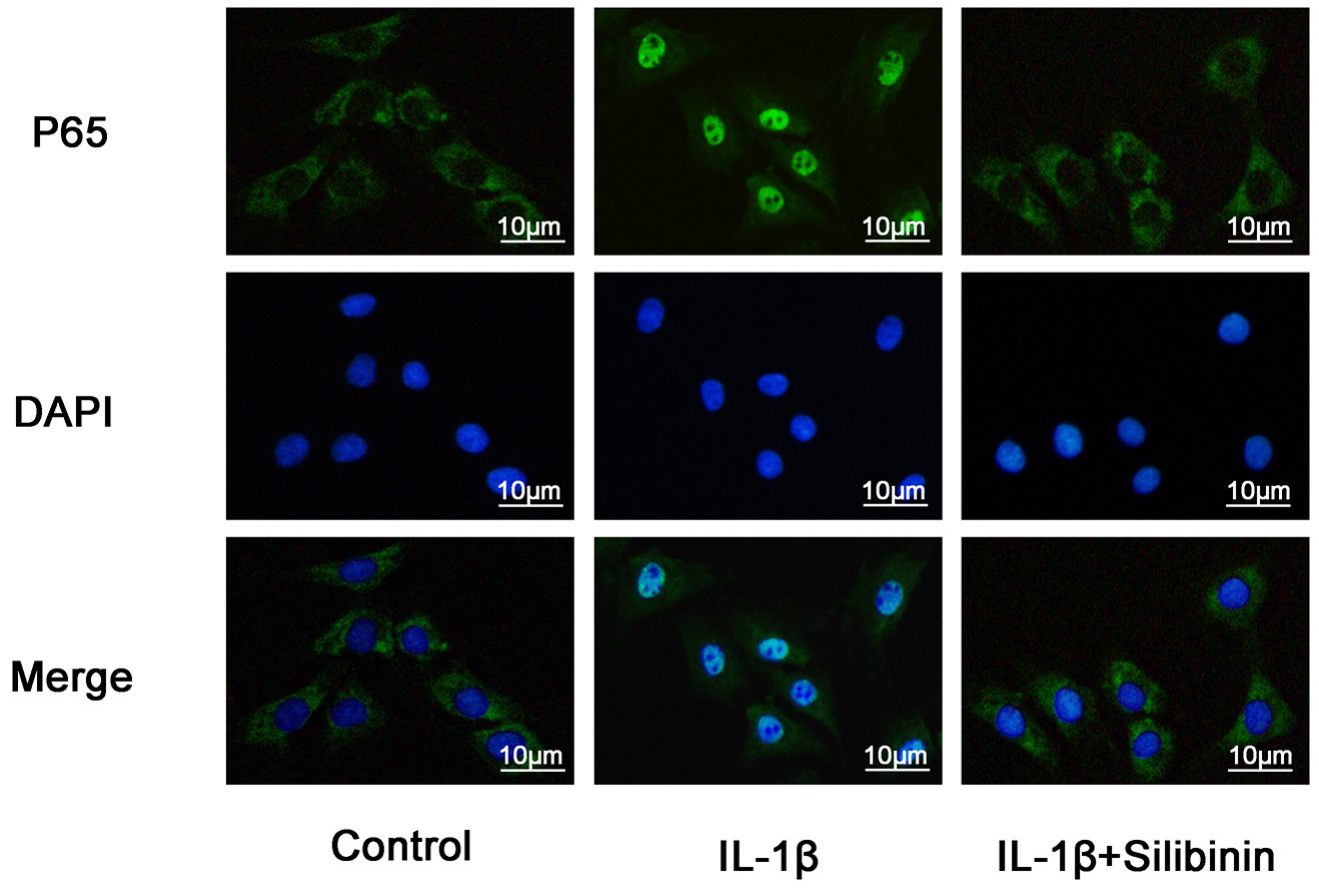
Effect of silibinin on IL-1β-induced nuclear translocation of NF-κB p65 in human OA Chondrocytes Chondrocytes remained unstimulated as control or were treated with IL-1β alone for 30min or co-treated with 10 ng/ml IL-1β and 50μM silibinin for 30min. The localization of p65 was visualized by immunofluorescence analysis as described in materials and methods. Data shown are representative of six independent experiments.

The effect of silibinin on NF-κB p65 nuclear translocation was examined by immunofluorescence in chondrocytes in response to NF-κB activation by IL-1β. Chondrocytes remained not stimulated or treated with IL-1β alone for 30 min or were co-treated with IL-1β and 50 μM silibinin for 30 min. Control chondrocytes showed the labeling of p65 mostly restricted in cytoplasm. Once IL-1β stimulated, chondrocytes revealed clear and enhanced nuclear staining for p65 and indicated nuclear translocation of the NF-κB p65 subunit. Nevertheless, silibinin resulted in inhibiting translocation of the p65 subunits into the nuclei and showed a decrease in activation of NF-κB (Figure 7). These immunofluorescent findings suggested silibinin is able to inhibit the nuclear translocation of NF-κB p65 in IL-1β-stimulated chondrocytes, which keeps consistent with the inhibitory effect of silibinin on NF-κB observed by Western blot.

### Effect of silibinin on IL-1β-induced PI3K/AKT phosphorylation in human OA chondrocytes

**Figure 8:**
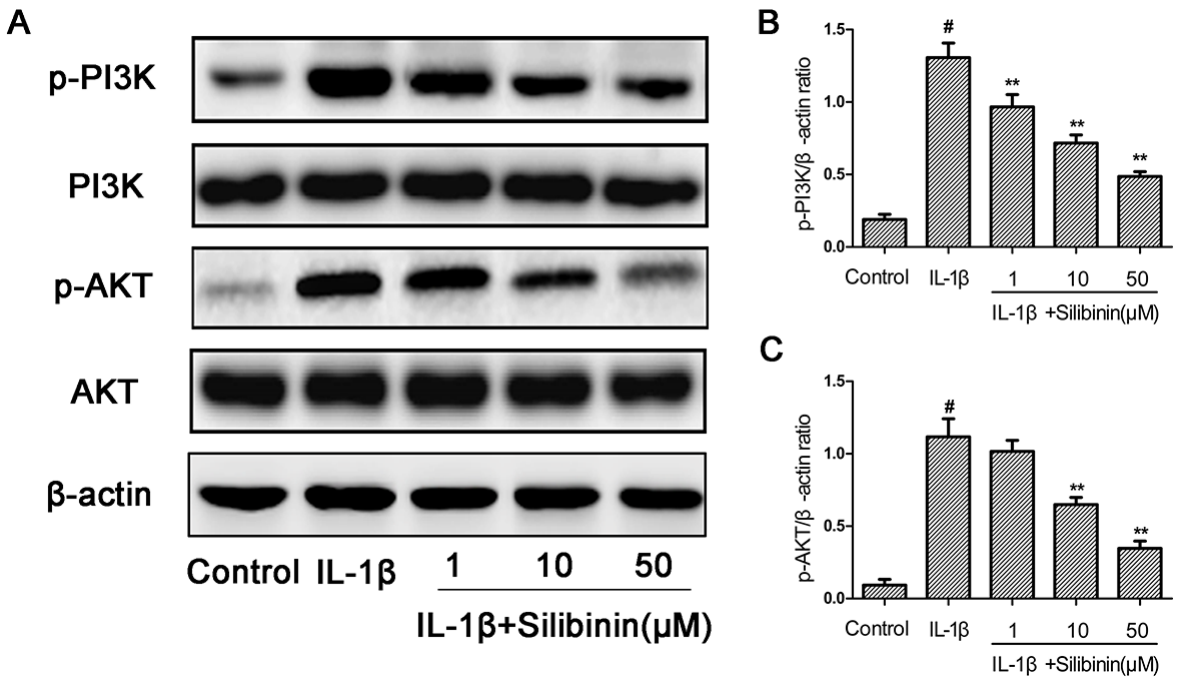
Effect of silibinin on IL-1β-induced PI3K/AKT phosphorylation in human OA Chondrocytes Chondrocytes were pretreated with silibinin (1, 10, 50 μM) for 2h, followed by stimulation with or without IL-1β (10 ng/ml) for 1 h. The protein expression levels of PI3K, p-PI3K, AKT and p-AKT were determined by Western blot and quantification analysis **(A-C)**. The values are mean ± SD. ^#^p < 0.05 compared with control group, ^*^p < 0.05, ^**^p< 0.01 compared with IL-1β group. n=6.

To further investigate the anti-inflammatory mechanism of silibinin, the effects of silibinin on IL-1β-induced PI3K/AKT phosphorylation were detected by Western blot analysis. As shown in Figure 8, IL-1β significantly increased the phosphorylation of PI3K and AKT compared to the control group. However, treatment of silibinin dose-dependently suppressed IL-1β-induced PI3K/AKT phosphorylation.

### Effect of silibinin on destabilization of the medial meniscus (DMM)-induced OA in mice

**Figure 9:**
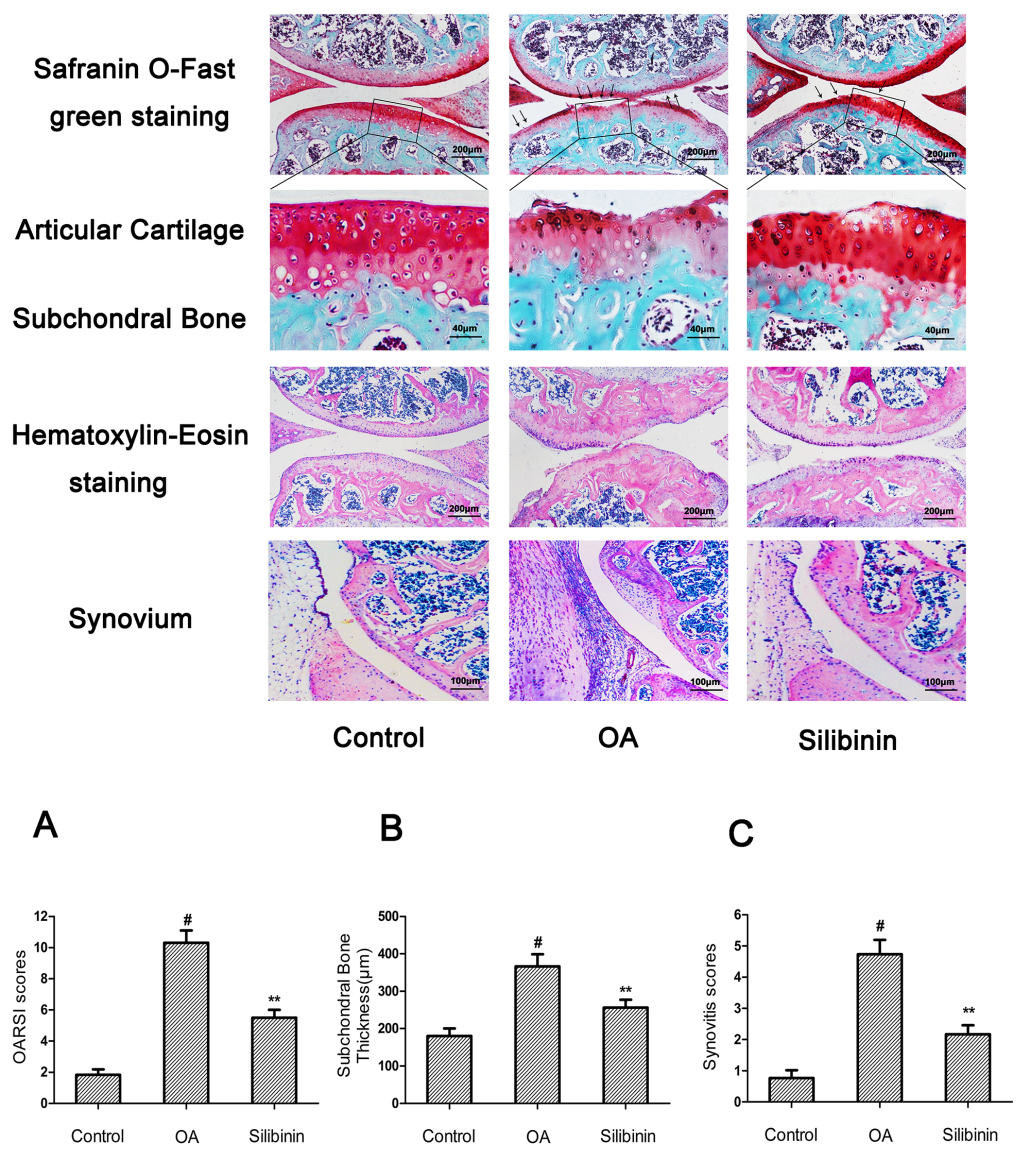
Silibinin attenuated the cartilage destruction, subchondral bone thickness and synovitis in mice OA models Mice were randomly divided into three groups: sham control group, OA group and silibinin group. Sham control group underwent sham operation, OA group and silibinin group were subjected to surgery for establishing OA models. After surgery, silibinin group received a gavage of silibinin (200 mg/kg) daily while OA group received a gavage of vehicle (0.5%CMC-Na) for 8 weeks. Histological analysis of OA was evaluated by Safranin O staining, Hematoxylin-Eosin staining, Osteoarthritis Research Society International (OARSI) scores **(A)**, subchondral bone plate thickness **(B)** and synovitis scores **(C)**. Lower panels show amplifications of upper black panel insets. Black arrows show cartilage destruction. The values are mean ± SD. ^#^p<0.05 compared with sham control group, ^*^p <0.05 compared with OA group. n = 10 for each group. Representative histologic images are shown.

**Figure 10:**
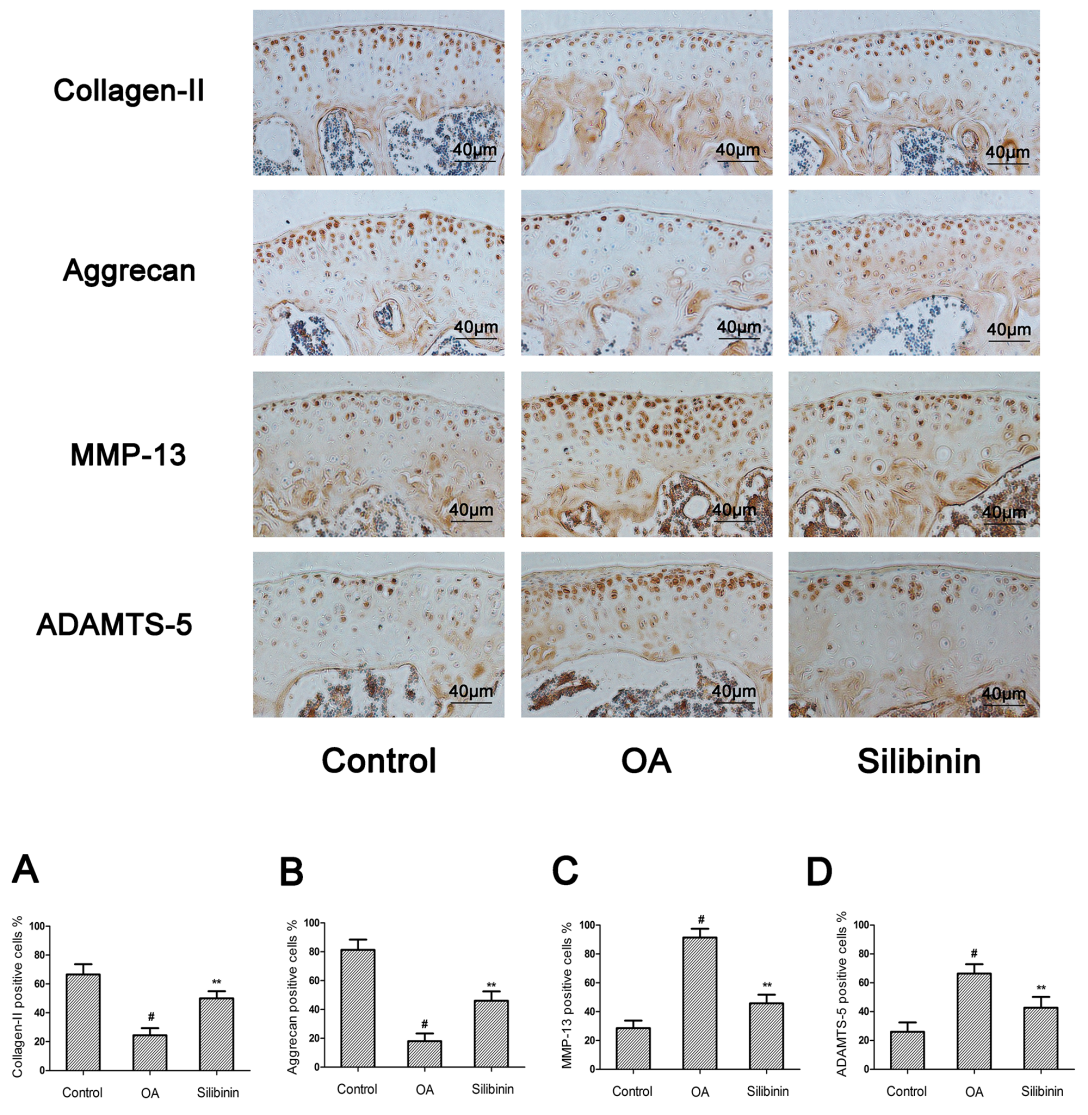
Effect of silibinin on cartilage matrix degradation in mice OA models Mice were randomly divided into three groups: sham control group, OA group and silibinin group. Sham control group underwent sham operation, OA group and silibinin group were subjected to surgery for establishing OA models. After surgery, silibinin group received a gavage of silibinin (200 mg/kg) daily while OA group received a gavage of vehicle (0.5%CMC-Na) for 8 weeks. Immunohistochemistry of collagen-II, aggrecan, MMP-13 and ADAMTS-5 were assessed to the effect of silibinin on cartilage matrix degradation in mice OA models. Quantifcation of collagen-II, aggrecan, MMP-13 and ADAMTS-5-positive cells in cartilage samples **(A-D)**. The values are mean ± SD. ^#^p<0.05 compared with sham control group, ^*^p <0.05 and ^**^p< 0.01 compared with OA group. n = 10 for each group. Representative histologic images are shown.

To evaluate whether silibinin has preventive effect against the induction and progression of osteoarthritis *in vivo*, DMM OA models were established in mice, followed by a gavage of silibinin (200mg/kg) daily for 8 weeks after surgery. As shown in Figure 9, the Safranin-O staining and Hematoxylin-Eosin staining showed the cartilage surface was smooth and intact in the sham control group. However, OA group exhibited cartilage superficial destruction, massive proteoglycan loss and apparent hypocellularity compared to the sham control group. In contrast, treatment of silibinin remarkably protected the structure of articular cartilage and maintained the proteoglycan in cartilage. On the basis of the results of Safranin O staining, the OARSI scores of OA group were manifestly higher (10.30±0.46) than the sham control group (1.83±0.20). On the contrary, silibinin group showed transparently lower OARSI scores (5.50±0.29) than OA group. Moreover, it was found that silibinin obviously lowered the subchondral bone thickness (Figure 9B) and ameliorated the inflammatory changes in synovium (Figure 9C) compared to OA group. We further investigated the expression of collagen-II, aggrecan, MMP-13 and ADAMTS-5 in cartilage matrix using immunohistochemistry. As shown in Figure 10, quantitative analysis demonstrated that OA group showed less number of collagen-II and aggrecan positive cells than the sham control group. However, treatment of silibinin highly increased the number of collagen-II and aggrecan positive cells in comparison with OA group (Figure 10A-10B). On the other side, OA group visibly increased the number of MMP-13 and ADAMTS-5 positive cells compared to the sham control group, which was observably decreased by the treatment of silibinin (Figure 10C-10D). Taken together, these results indicate that silibinin protected cartilage against degradation partly by inhibiting MMP13 and ADAMTS-5 and enhancing collagen-II and aggrecan expression.

## DISCUSSION

Currently, treatment of OA has mainly aimed to relieve the chief complaint of OA patients, including joint pain, swelling and muscle tightness. Present pharmacological treatments for OA such as nonsteroidal anti-inflammatory drugs (NSAIDs) are only temporarily effective and always bring about numerous serious side effects [18]. Therefore, there is a considerable need to seek safe and more effective therapeutic strategies for the treatment of OA. Accumulating evidence suggests compounds extracted from natural plants have become the potential and reasonable agents of choices for OA patients because of their strong anti-inflammatory activities and less toxicity [9]. Silibinin, a polyphenolic flavonoid extracted from seeds of *Silybum marianum*, has been reported to have potent anti-inflammatory effects [15]. In the present study, we demonstrated the effects of silibinin (0-50μg/ml) on human OA chondrocytes were not attributable to cytotoxic effects. We found that silibinin significantly inhibited the IL-1β-induced inflammatory response, including the expression of NO, PGE2, TNF-α, IL-6, iNOs, COX-2, MMP-1, MMP-3, MMP-13, ADAMTS-4 and ADAMTS-5 as well as the degradation of collagen-II and aggrecan in human OA chondrocytes. Besides, our results showed that silibinin remarkably blocked the IL-1β-induced NF-κB activation and PI3K/AKT phosphorylation. Furthermore, silibinin exerted a protective effect against cartilage degradation in DMM-induced OA in mice.

Inflammatory mediators such as NO and PGE2 have long been demonstrated to play critical roles in OA in that they accelerate the development of OA by inducing MMPs synthesis and inhibiting collagen and proteoglycan synthesis in chondrocytes [19, 20]. NO is an inflammatory mediator which is produced by the nitric oxide synthase (NOS) family of enzymes [21]. It has been reported that NO promotes the production and activation of MMPs as well as other inflammatory cytokines in OA [22]. PGE2 is also an inflammatory mediator that is elevated by cyclooxygenase-2 (COX-2), is involved in the degeneration of cartilage during the pathophysiology of OA due to its potent excitation on MMPs and inhibition of extracellular matrix (ECM) synthesis in cartilage [23]. Previous studies have shown that inhibition of the production of inflammatory mediators such as NO and PGE2 were capable of attenuating the progression of OA [24]. In this study, we demonstrated that silibinin significantly inhibited IL-1β-induced NO and PGE2 production as well as iNOS and COX-2 expression at the mRNA and protein levels in human OA chondrocytes. Our findings agree with the previous studies which have reported that silibinin dose-dependently suppressed the PGE2 production and COX-2 expression in LPS-stimulated RAW 264.7 cells [16] and administration of silibinin significantly decreased the levels of NO and the over expression of iNOS in the kidney tissue of nephrotoxicity rats induced by arsenic [25]. TNF-α and IL-6 play pivotal roles in many inflammatory diseases including OA. They could activate macrophages, which subsequently synthesize pro-inflammatory chemokines to maintain inflammation in the development of OA [26]. In our study, we found that induction of TNF-α and IL-6 production by IL-1β was abolished by silibinin. The results are consistent with Khan et al.’s results [27]. They found that pretreatment with silibinin significantly inhibited 12-O-tetradecanoyl-phorbol-13-acetate (TPA)-induced cutaneous TNF-α and IL-6 production in Swiss albino mice. Taken together, our results suggest the feasibility of silibinin in the treatment of OA on the basis of the strong inhibition of the overproduction of NO, PGE2, TNF-α and IL-6 as well as the overexpression of iNOS and COX-2 in the progression of OA.

MMPs comprise a family of enzymes that promote the cartilage ECM turnover and breakdown in the pathophysiology of OA. Among all MMPs, MMP-13 has been shown to play a major role in the progression of OA since it forcefully and irreversibly cleaves collagen-II which almost constitutes the main structure of the cartilage ECM and makes up the structural rigidity of cartilage [28, 29]. It has been demonstrated that selective inhibitors of MMP-13 provided a potential therapy that could slow the progression of OA [30]. MMP-1 and MMP-3 also have been reported to break down a number of ECM structural proteins such as collagen-II and proteoglycans. Evidence is accumulating for the importance of the ADAMTS family of proteins in cartilage degradation in OA, especially ADAMTS-4 and ADAMTS-5 [31]. It has been demonstrated that ADAMTS-4 and ADAMTS-5 are considered as the primary aggrecanases responsible for the cleavage of aggrecan in the pathogenesis of the OA, which has made them potential therapeutic targets for treating OA [32]. Inhibition of ADAMTS-5 by small interfering RNA (siRNA) decreased aggrecan loss from human OA cartilage explants [33]. Interestingly, our results showed that the up-regulated MMP-1, MMP−3, MMP−13, ADAMTS-4 and ADAMTS-5 expression in IL-1β-stimulated human OA chondrocytes were dramatically decreased by silibinin in a dose-dependent manner. Accordingly, we speculated that silibinin may exert its chondroprotective effects by blocking the expression and activation of MMPs and ADAMTS in the pathogenesis of OA.

Collagen-II and aggrecan constitute the major structure of the ECM and maintain the normal physiological function of the cartilage. Loss of collagen-II and aggrecan contributes to accelerating the progress of OA. Therefore, inhibiting degradation of collagen-II and aggrecan may a new therapeutic choice for OA. In our study, silibinin exhibited a potent inhibition on the down-regulation of aggrecan and collagen-II mRNA as well as collagen-II protein expression in human OA chondrocytes, which suggests that silibinin suppresses cartilage degradation may through enhancing the expression of aggrecan and collagen-II.

**Figure 11:**
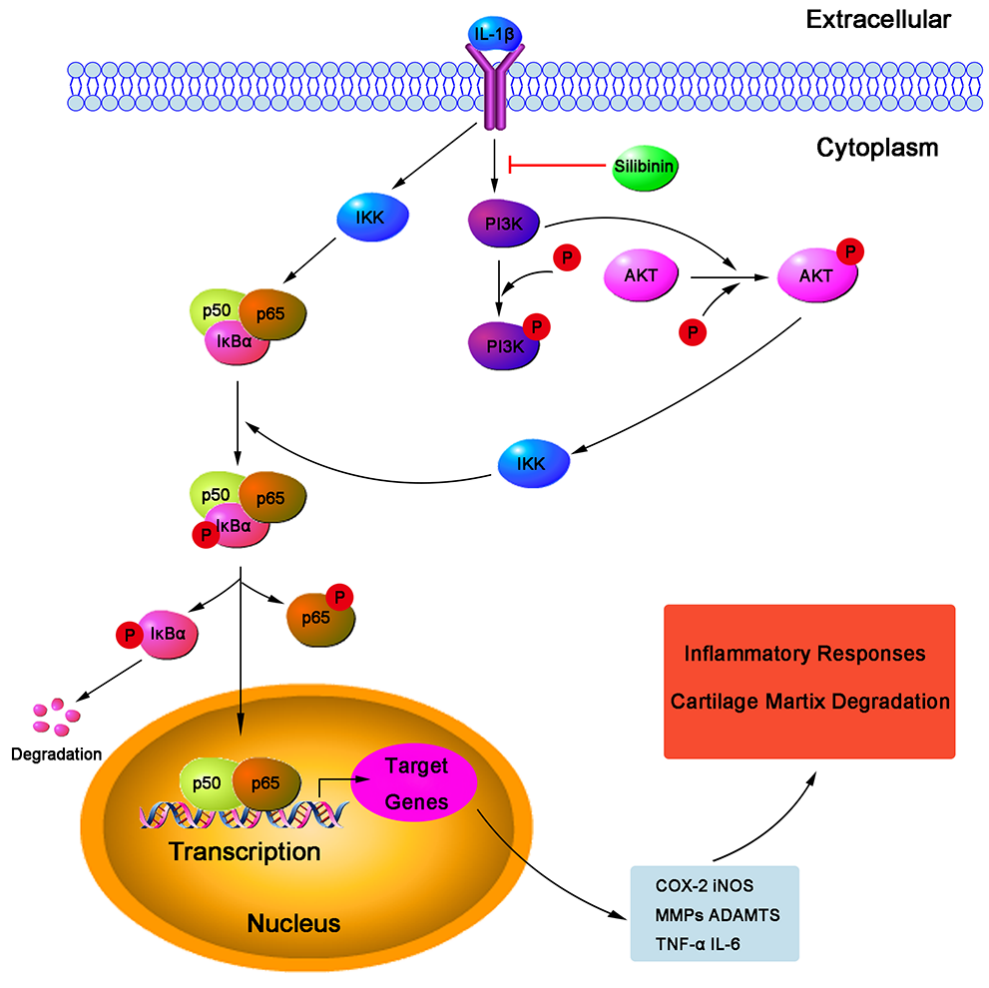
Working model for the inhibitory effects of silibinin on IL-1β-induced PI3K/AKT phosphorylation and NF-κB activation resulted in destruction of cartilage in human OA chondrocytes *in vitro*

NF-kB is one of the inflammatory cytokine induced transcription factors with a vital role in regulating the expression of iNOS, COX-2, TNF-α, IL-6, MMPs and other cytokines as well as the regulation of plenty of inducible inflammatory genes in OA [34]. Normally, NF-kB is present in the cytoplasm in an inactive form combined with the inhibitory subunit IκBα. Once activated by IL-1β, IκBα is phosphorylated and degraded and NF-κB p65 translocates from the cytoplasm to the nucleus to trigger the expression of inflammation-related genes, including iNOS, COX-2, TNF-α, IL-6, MMPs and ADAMTS [35, 36]. Hence, blocking the activity of NF-kB may be effective therapeutically in OA. Previous reports showed that suppression of NF-κB activation had the ability to attenuate the progression of OA [37]. In addition, a study also reported that NF-κB p65-specific siRNA inhibited the expression of COX-2, iNOs and MMP-9 in IL-1β-induced chondrocytes [38]. Therefore, in our study, we also investigated molecular mechanisms by which silibinin inhibited inflammatory mediators in response to IL-1β in human OA chondrocytes. We found that silibinin significantly inhibited IL-1β-induced NF-κB activation and IκBα degradation in human OA chondrocytes. Furthermore, silibinin reversed the translocation of NF-κB p65 from cytoplasm to nucleus when detected by immunofluorescence staining. Our results are partly supported by Yun et al. who found that silibinin inhibited the production of pro-inflammatory cytokines through inhibition of NF-κB signaling pathway in HMC-1 human mast cells [14]. Besides, silibinin suppressed the nuclear translocation of NF-κB and phosphorylation of IκBα in lung tissues of allergic mice [17]. The intracellular signaling pathway PI3K/Akt has also been demonstrated to be involved in both cellular and ECM alterations in OA pathogenesis [39]. Activation of the PI3K/Akt pathway can increase MMPs production by chondrocytes via its multiple downstream target proteins such as NF-κB. Moreover, inhibition of the PI3K/Akt pathway has been considered as an option for the treatment of OA. Indeed, inhibition of p-Akt and NF-κB by curcumin decreased IL-1β stimulating MMPs secretion and COX-2 expression in chondrocytes [40]. In our study, we found that silibinin greatly suppressed IL-1β-induced PI3K/Akt phosphorylation in human OA chondrocytes, which was partly supported by the previous study showing that silibinin exerted inhibtory effects on 12-O-tetradecanoylphorbol-13-acetate-induced skin inflammation via inhibiting PI3K/Akt signaling pathway [41]. Taken together, the previous studies together with our findings suggest that silibinin inhibited IL-1β-induced inflammatory response through suppressing the PI3K/Akt phosphorylation and NF-kB activation in human OA chondrocytes and the underlying mechanisms was illustrated specifically in Figure 11. However, further studies are needed to clarify the exact mechanism of the regulation of silibinin on the inflammatory process in human chondrocytes.

In addition to the vitro study, we used the DMM model to assess the anti-inflammatory effect of silibinin *in vivo*. DMM model has been widely applied to evaluate the efficacy of drugs in the treatment of OA because of its mechanical instability [42]. There is growing evidence suggests cartilage destruction, subchondral bone remodeling and synovitis are actively involved in the complex initiation and development of OA [43, 44]. As a result, agents that specifically block the mechanisms associated with cartilage destruction, subchondral bone remodeling and synovitis may be beneficial to develop targeted therapy for OA. In the current study, our results showed that the mice in OA group exhibited severe cartilage destruction and extensive proteoglycan loss, which was obviously alleviated by treatment of silibinin. Moreover, silibinin reduced the OARSI scores and subchondral bone plate thickness and ameliorated the severity of synovitis in mice OA, indicating that silibinin had the ability to attenuate the progression of OA. Furthermore, treatment of silibinin significantly decreased the expression of MMP-13 and ADAMTS-5 and increased the expression of collagen-II and aggrecan in mice OA. These results along with the vitro findings provide the evidence that silibinin possesses anti-inflammatory effects in OA both *in vitro* and *in vivo*. However, in this study, we didn’t evaluate the potential toxicity *in vivo* study and the exact mechanism of the regulation of silibinin on the inflammatory response *in vitro* has not been Figured out. This was a limitation of our research. Further work is needed to investigate the potential toxicity *in vivo* and clarify the exact mechanism of the regulation of silibinin on the inflammatory process in human chondrocytes.

## MATERIALS AND METHODS

### Chemicals and reagents

Silibinin (purity >98 %), recombinant human IL-1β, collagenase type II, Safranin-O/Fast Green and dimethylsulfoxide (DMSO) were obtained from Sigma Chemical Co. (St. Louis, MO, USA). Cell-Counting Kit-8 (CCK-8) was purchased from Dojindo (Kumamoto, Japan). Primary antibodies against COX-2, iNOS, MMP-1, MMP-3, MMP-13, ADAMTS-4 and ADAMTS-5 were purchased from Abcam (Cambridge, MA, USA). Primary antibodies against p65, p-p65, IkBα, p-IkBα, PI3K, p-PI3K, Akt and p-Akt were purchased from Cell Signaling Techonology (Beverly, MA, USA). Goat anti-rabbit and goat anti-mouse horseradish peroxidase conjugates were purchased from Bio-Rad Laboratories (Calif., USA). Fetal bovine serum (FBS), bovine serum albumin (BSA), Dulbecco’s modified Eagle’s medium (DMEM)/Ham’s F12 medium and 0.25% trypsin-ethylendiaminetetraacetic acid (trypsin–EDTA) were purchased from Gibco (Life Technologies Corp. Carlsbad, Calif., USA). TRIzol reagent was purchased from Invitrogen (Carlsbad, Calif., USA). QuantiTect Reverse Transcription kit was purchased from Qiagen (Valencia, CA). SYBR Green Master Mix was purchased from Bio-Rad Laboratories (Calif., USA). ELISA kits of PGE2, TNF-α and IL-6 were purchased from R&D systems (Minneapolis, MN, USA). Griess reagent was purchased from Beyotime Institute of Biotechnology (Shanghai, China). All other chemicals were of reagent grade.

### Primary human chondrocytes isolation and culture

Articular cartilage samples collection was according to the terms of the Medical Ethical Committee of the Second Affiliated Hospital, Wenzhou Medical University and following the guidelines of the Declaration of Helsinki and Tokyo. OA human cartilage tissues were obtained from ten OA patients (age 57 ± 9) who underwent total knee replacement surgery at the Second Affiliated Hospital of Wenzhou Medical University. Osteoarthritis was diagnosed according to the Diagnostic and Therapeutic Criteria Committee of the American Rheumatism Association [45]. Full ethical consent was obtained from all patients. Primary chondrocytes were isolated from articular cartilage as described previously [46]. Cartilage pieces were digested with 0.2% collagenase type II for 5 h at 37°C. Then centrifuged at 1000 rpm for 5 min and the supernatant was discarded. The inner cell mass was obtained and suspended in DMEM/F12 with 10% FBS and 1% antibiotic mixture (Penicillin and Streptomycin). Finally, cells were plated at a density of 1 × 10^5^ cells/ml in 6-well plate and incubated in a humidified atmosphere of 5% CO_2_ at 37°C. The media were changed every 2-3 days. Cells were passaged when at 80 to 90% confluence using 0.25% trypsin–EDTA solution. Only passage 1 to 2 were used in our study to avoid the phenotype loss.

### Cell viability

The effects of silibinin on the viability of cells were assessed by CCK-8 assay according to the manufacturer’s instructions. Human OA chondrocytes were cultured in 96-well plates at a density of 5 × 10^3^ cells per well for 24 h. Then cells were pretreated with different concentrations (0, 1, 10 and 50 μM) of silibinin for 24 h and 48h. After that, 10μL CCK-8 was added to each well and incubated at 37°C for 4 h. The optical density was read at a wavelength of 450 nm with a microplate reader (Leica Microsystems, Germany).

### Measurement of NO, PGE2, TNF-α and IL-6

The NO levels in the culture medium were determined by Griess reaction as previously described [47]. The levels of PGE2, TNF-α and IL-6 in the culture medium were investigated using commercial ELISA kits according to the manufacturer’s instructions (R&D Systems, Minneapoils, MN). All assays were performed in duplicate.

### Quantitative real-time polymerase chain reaction

Total RNA was isolated from chondrocytes using TRIzol reagent according to the manufacturer’s instructions. Its concentration was determined spectrophotometrically at 260 nm (Thermo Scientific NanoDrop 2000). The A260/A280 ratio was calculated to verify quality and purity. First-strand cDNA was synthesized using 1μg of total RNA and the QuantiTect Reverse Transcription kit. Quantitative real-time PCR(qPCR) was performed using CFX96Real-TimePCR System (Bio-Rad Laboratories, California, USA), under the following conditions: 10 min 95°C, followed by 40 cycles of 15 s 95°C and 1 min 60°C. The reaction was performed in a total volume of 10 μL, containing 4.5μL diluted cDNA, 0.25 μL forward primer, 0.25μL reverse primer and 5 μL SYBR Green Master Mix. The level of target mRNA was normalized to the level of GAPDH and compared with control. Data were analyzed using 2^−ΔΔCT^ method [48]. Each gene analysis was performed in triplicate. Primer’s sequences of the targeted genes were listed in Table 1.

**Table 1:** Primer sequences used in qRT-PCR

Gene	Forward primer	Reverse primer	Origin
COX-2	5′-GAGAGATGTATCCTCCCACAGTCA-3′	5′-GACCAGGCACCAGACCAAAG-3′	Human
iNOS	5′-CCTTACGAGGCGAAGAAGGACAG-3′	5′-CAGTTTGAGAGAGGAGGCTCCG-3′	Human
MMP-1	5′-GGGAATAAGTACTGGGCTGTTCAG-3′	5′-CCTCAGAAAGAGCAGCATCGATATG-3′	Human
MMP-3	5′-CTGGCCTGCTGGCTCATGCTT-3′	5′-GCAGGGTCCTTGGAGTGGTCA-3′	Human
MMP-13	5′-CCAGAACTTCCCAACCAT-3′	5′-ACCCTCCATAATGTCATACC-3′	Human
ADAMTS-4	5′-CCTGGCAAGGACTATGATGCTGA-3′	5′-GGGCGAGTGTTTGGTCTGG-3′	Human
ADAMTS-5	5′-GCAGAACATCGACCAACTCTACTC-3′	5′-CCAGCAATGCCCACCGAAC-3′	Human
Collagen-II	5′-CTCAAGTCGCTGAACAACCA-3′	5′-GTCTCCGCTCTTCCACTCTG-3′	Human
Aggrecan	5′-AAGTGCTATGCTGGCTGGTT-3′	5′-GGTCTGGTTGGGGTAGAGGT-3′	Human
GAPDH	5′-TCTCCTCTGACTTCAACAGCGAC-3′	5′-CCCTGTTGCTGTAGCCAAATTC-3′	Human

### Western blot analysis

The proteins were extracted from chondrocytes with RIPA lysis buffer. Lysates were sonicated on ice and centrifuged at 12,000 rpm for 30 min at 4°C. The protein concentration of the supernatant was determined using the BCA protein assay kit. Equal amounts of protein (40 μg) were separated by 12% SDS-PAGE and transferred to PVDF membranes. Membranes were incubated with blocking buffer 5% non-fat milk or 3-5% bovine serum albumin (BSA) (for phosphorylated protein) in TBS containing 0.1% Tween-20 (TBST) for 2 h at room temperature and then probed with the primary antibodies against COX-2, iNOS, MMP-1, MMP-3, MMP-13, ADAMTS-4 and ADAMTS-5, p65, p-p65, IkBα, p-IkBα, PI3K, p-PI3K, Akt and p-Akt (dilution 1:1000) overnight at 4°C. After washing three times with TBS containing 0.1% Tween-20 for 5 min, the membranes were incubated with HRP-conjugated secondary antibodies (1:3000) for 2 h. Finally membranes were detected by Enhanced Chemiluminescence (ECL) kit and quantified by the Quantity ONE (Bio-Rad, Hercules, CA, USA) software. β-actin was used as an internal control.

### Immunofluorescence

Chondrocytes were seeded on 6-well plates on glass coverslips and incubated for 24 h. Glass coverslips with chondrocyte monolayers were rinsed three times in PBS. Then cells were fixed with 4% paraformaldehyde for 15 min at room temperature and rinsed with PBS again. Cells and nuclear membranes were permeabilized with 0.1% Triton X-100 in for 5 min at room temperature. Later, cells were overlaid with 5% protease-free BSA for 1h at room temperature, rinsed with PBS and incubated with primary antibody against collagen-II and p65 (1:200) at 4°C overnight. After washing with PBS, cells were incubated with fluorescein-conjugated goat anti-rabbit IgG antibody (1:500) for 1 h at room temperature. Finally, cells were washed three times with PBS and mounted in medium containing DAPI (Invitrogen). Slides were viewed with confocal laser scanning microscope Leica TCS SP8 using Leica Application Suite X software version 3.3.0 (Leica Microsystems, Germany).

### Transient transfection and luciferase activity assay

1μg of NF-κB promoter/luciferase DNA (Stratagene, Santa Clara, CA, USA) along with 20 ng of control pRL-TK DNA was transiently transfected into 1×10^5^ chondrocytes/well in a 6-well plate using lipofectamine/plus reagents for 24 h. Cells pretreated with silibinin (1,10,50μM) were stimulated with IL-1β (10 ng/ml) for 2h. Each well was then washed twice with ice-cold PBS and harvested in 100 μl of lysis buffer (0.5 mM HEPES pH 7.8, 1 % Triton N-101, 1 mM CaCl2, and 1 mM MgCl2) and used for the measurement of luciferase activity using a dual-luciferase assay kit. Luminescence was measured on a top counter microplate scintillation and luminescence counter in single photon counting mode for 0.1 min/well, following a 5 min adaptation in the dark. The luciferase activity was normalized with expression of control pRL-TK.

### Mice OA models

Eight-week-old C57BL/6 male wild-type (WT) mice were purchased from Animal Center of Chinese Academy of Sciences, Shanghai, China. The protocol for animal care and use conformed to the Guide for the Care and Use of Laboratory Animals of the National Institutes of Health and was approved by the Animal Care and Use Committee of Wenzhou Medical University. The experimental mice were subjected to surgically induced OA by destabilization of the medial meniscus (DMM) as previously described [49]. Briefly, after anaesthesia with peritoneal injection of 1% pentobarbital sodium, the cranial attachment of the medial meniscus to the tibial plateau (medial meniscotibial ligament) of the right knee was transected with a microsurgical knife. The lateral meniscotibial ligament was identified and protected during the surgery. A sham operation, consisting of an arthrotomy without the transaction of medial meniscotibial ligament, was also performed in the right knee joint of mice in sham control group.

### Experimental animal design

The mice were randomly divided into three groups of 10 mice to establish a sham control group (control), an osteoarthritis group (OA) and an osteoarthritis treated with silibinin group (silibinin). Mice OA models were made by DMM surgery. Silibinin was dissolved in 0.5% sodium carboxymethyl cellulose (0.5%CMC-Na). Mice in sham control group were made by sham operation. Mice in silibinin group received a gavage of silibinin (200mg/kg) daily for 8 weeks after surgery while mice in OA group received a gavage of vehicle (0.5%CMC-Na). Food and water were available ad libitum. Mice were maintained under a constant temperature of 20±2C°, a relative humidity of 50%±10%, and a 12 h light/dark cycle. All animals were sacrificed at 8 weeks after surgery. Knee joint tissues were collected for further evaluation.

### Histological assessment and immunohistochemistry

Collected joint samples were fixed in 4% paraformaldehyde for 24 hours at 4°C and decalcified in 10% EDTA solution at 4°C for two weeks. After that, the samples were dehydrated through an alcohol gradient, cleared, and embedded in paraffin blocks. Frontal serial sections (5 μm thick) across entire joints were obtained and 10 slides per joint at every 50 μm were selected and stained with Safranin-O/Fast Green and Hematoxylin-Eosin to assess cartilage destruction. The stained sections were photographed digitally under a microscope. To determine the extent of cartilage degeneration, we used multiple separate scoring systems for articular cartilage destruction, synovitis and subchondral bone thickness. The destruction of articular cartilage was graded using the Osteoarthritis Research Society International (OARSI) scoring system for medial femoral condyle and medial tibial plateau [50]. Then we used a summed OARSI score (0-12) from medial femoral condyle and medial tibial plateau to evaluate the degree of articular cartilage destruction. The severity of synovitis was graded using a scoring system which was previously described [51]. We applied AxioVision software to measure the thickness of the medial subchondral bone plate according to Safranin O stained sections. Mouse cartilage sections (5μm) were prepared using a freezing microtome. After overnight incubation at 4°C with primary antibodies for collagen-II, aggrecan, MMP-13 and ADAMTS-5, the histological sections were incubated with secondary antibodies (Beyotime Institute of Biotechnology Inc., Jiangsu, China) for 2 h at room temperature. The DAB substrate system (Zsbio, Beijing, China) was used for color development. Hematoxylin staining was utilized to reveal the nuclei of the cells. The number of positively stained cells on the entire articular surface per specimen was counted and the percentage positive cells was calculated [52].

### Statistical analysis

All Data are presented as mean ± standard deviation (SD). Comparisons between groups were analyzed using SPSS 17.0 software via ANOVA followed by Dunnett’s test. A ^*^p<0.05, ^**^p<0.01, ^***^p<0.001 and ^****^p<0.0001 was considered statistically significant.

## CONCLUSION

In conclusion, the present study demonstrated that silibinin inhibited IL-1β-induced inflammation as well as PI3K/Akt phosphorylation and NF-κB activation in human OA chondrocytes. Furthermore, silibinin exerted a protective effect against cartilage degradation in DMM-induced OA in mice. Taken together, these findings suggest that silibinin may be a potential therapeutic choice in the treatment of OA in the future.

## ACKNOWLEDGMENTS AND FUNDING

## Abbreviations

OA: osteoarthritis
ECM: extracellular matrix
TNF-α: tumor necrosis factor-alpha
IL-1β: interleukin-1β
IL-6: interleukin-6
COX-2: cyclooxygenase 2
NO: nitric oxide
PGE2: prostaglandin E2
iNOS: inducible nitric oxide synthase
MMPs: matrix metalloproteinase
ADAMTS: a disintegrin and metalloproteinase with thrombospondin motifs
NSAIDs: non-steroidal anti-inflammatory drugs
NF-κB: nuclear factor-kappa B
LPS: lipopolysaccharides
qPCR: quantitative real-time polymerase chain reaction
DMM: destabilization of the medial meniscus
OARSI: Osteoarthritis Research Society International
PI3K/AKT: phosphatidylinositol 3 kinase/ protein kinase B
SD: standard deviation
